# Efficacy and Safety of Tenofovir Alafenamide Fumarate and Tenofovir Disoproxil Fumarate for Preventing Mother-to-Child Transmission of Hepatitis B in Treatment-Naïve mothers: An Observational Study

**DOI:** 10.1186/s12985-026-03074-9

**Published:** 2026-02-02

**Authors:** Xueyao Yang, Jun Chen, Lihua Duan, Xuexuan Li, Weiting Cheng, Ying Chen, Yan Huang, Zebing Huang

**Affiliations:** 1 Department of Infectious Diseases, Xiangya Hospital, Central South University, Changsha, 410008 China; 2 Key Laboratory of Viral Hepatitis of Hunan Province, Xiangya Hospital, Central South University, Xiangya Road 87, Kaifu District, Changsha, 410008 China; 3 National Clinical Research Center for Geriatric Disease, Xiangya Hospital, Central South University, Changsha, 410008 China; 4 Xiangya Hospital, Central South University, Changsha, China 87 xiangya road, 410008

**Keywords:** Retrospective study, Chronic hepatitis B, Nucleos(t)ide analogue, Mother-to-child transmission, Tenofovir, Tenofovir alafenamide fumarate

## Abstract

**Background:**

Mother-to-child transmission (MTCT) is the primary cause of hepatitis B virus (HBV) infection. Antiviral therapy is crucial to reduce MTCT for pregnant women with high viremia. Tenofovir alafenamide (TAF) and tenofovir disoproxil fumarate (TDF) are the first-line antiviral drugs for hepatitis B. This study aimed to evaluate the effectiveness and safety of TAF and TDF in preventing HBV MTCT among treatment-naïve mothers.

**Methods:**

A total of 290 pregnant women with HBsAg positive for > 6 months, and HBV DNA ≥ 2 × 10^5 IU/ml or HBeAg positive were enrolled. Mothers received either TDF or TAF therapy and newborns received hepatitis B immunoglobulin and recombinant yeast hepatitis B vaccine. MTCT was evaluated during a one-year follow-up period after birth.

**Results:**

290 pregnant women (185 with TDF therapy and 105 with TAF therapy) and 296 newborns (190 in TDF group and 106 in TAF group) were included. Both TDF and TAF effectively deceased HBV DNA levels with no significant difference, and the MTCT rates in TDF group and TAF group were similarly low (1.08% vs. 0.95%, *P* > 0.05). Moreover, no congenital malformations or growth and developmental abnormalities in newborns were observed in either group. However, serum creatinine was significantly higher and eGFR decreased significantly when treated by TDF, whereas TAF showed no significant renal effects.

**Conclusions:**

Both TAF and TDF are effective in preventing HBV MTCT, with comparable MTCT rates. However, TAF demonstrated superior renal safety, making it a preferable option for preventing HBV MTCT in treatment-naïve mothers.

## Introduction

Hepatitis B virus (HBV) infection is a global public health challenge that poses a significant threat to human health [1]. Mother-to-child transmission (MTCT) is the most common contributor to HBV infection globally and the leading cause for HBV infection chronicity [2]. Preventing MTCT is, therefore, a pivotal strategy in reducing the overall burden of HBV. While the administration of hepatitis B immune globulin (HBIG) and hepatitis B vaccine to newborns significantly reduces the risk of MTCT, it does not block all MTCT [3], particularly in newborns of mothers with high viral loads [4]. To achieve the World Health Organization (WHO) hepatisis elimilation targets by 2030, antiviral treatment for high viral load pregnant women is the key step in further minimizing MTCT.

Although lamivudine (LAM) and telbivudine (LdT) have demonstrated efficacy and safety in preventing HBV MTCT, their high rates of drug resistance have significantly limited their use for preventing MTCT [5–7]. Tenofovir disoproxil fumarate (TDF), a first-line antiviral drug for HBV MTCT prevention, has shown no resistance after three years of use and less than 1% resistance after six years [8, 9]. However, TDF had potential kidney damage and lowered neonatal bone mineral content [10, 11]. Additionally, small quantities of TDF have been detected in breast milk, raising questions about its impact on breastfeeding newborns.

Tenofovir alafenamide fumarate (TAF), as a new prodrug of tenofovir, features an amide bond addition to the TDF structure. The majority of TAF is hydrolyzed into tenofovir within liver cells in the context of HBV infection, exhibiting excellent liver targeting. Approved by the FDA in October 2016 for the treatment of chronic hepatitis B (CHB) in adults, TAF was also recommended as a primary treatment option in the 2017 European Association for the Study of the Liver (EASL) guidelines. Furthermore, TAF has also been considered for preventing HBV MTCT. Studies showed that TAF antiviral treatment for highly viremic mothers in late pregnancy effectively blocked MTCT without safety issues for childbirth or infant development [12, 13]. Moreover, TAF concentration was low in breast milk with negligible infant exposure [14].

However, in previous studies, they were not clarified whether the patients were experiencing antiviral drugs for the first time. This study aims to evaluate the efficacy and safety of TAF and TDF in preventing HBV MTCT among treatment-naïve mothers.

## Methods

### Study subjects

Pregnant women with HBV infection at the outpatient clinic of Infectious Disease Department or Obstetrics were enrolled from October 2015 to October 2023 in Xiangya Hospital. This study is a non-interventional study and does not involve patient privacy information, which was in compliance with the Declaration of Helsinki and was approved by the Xiangya Hospital Ethics Committee (Ethics Number: 202601001).

Inclusion criteria.

1. Maternal age: 20–40 years old;
2. HBsAg positive for > 6 months, and HBV-DNA ≥ 2 × 10^5 IU/ml or HBeAg positive;
3. TDF or TAF antiviral therapy during pregnancy;
4. No history of previous interferon and/or nucleos(t)ide analog therapy.

Exclusion criteria.

1. Patients with cirrhosis or hepatocellular carcinoma;
2. Patients with other viral hepatitis (Hepatitis A, Hepatitis C, Hepatitis D), autoimmune, or cholestatic liver diseases;
3. Patients with severe respiratory, circulatory, or neurological, and thyroid disease, etc.;
4. Patients with HIV or other severe immunodeficiency disorders;
5. Use of immunosuppressive, steroid, or cytotoxic drugs;
6. Signs of threatened miscarriage, pregnancy preservation therapy, or fetal abnormalities in early pregnancy.

## Study design

### Therapy and grouping

In this retrospective study, patients were divided into two groups: TAF (25 mg/d) group and TDF (300 mg/d) group. Patients voluntarily initiated antiviral therapy with either TDF or TAF during pregnancy after clinical consultation. After childbirth, mothers who started treatment primarily for the purpose of preventing mother-to-child transmission were recommended to end treatment, and mothers who started treatment meeting antiviral treatment criteria were recommended to continue treatment. However, the final decision to continue treatment remained with the patient based on consultation with their physician. Mothers who continued medication for over 1 month were classified as the postpartum continuation group, while those who stopped medication within 1 month were designated as the postpartum discontinuation group. All patients were informed about potential adverse effects during treatment.

Newborns received 100 IU of HBIG and 10 µg of recombinant yeast-derived hepatitis B vaccine within 12 h of birth, and received the vaccine at one month and six months of age.

### Efficacy assessment indicators

For pregnant women: normalization rate of alanine transaminase (ALT), aspartate aminotransferase (AST), HBV-DNA levels, and HBV DNA seroclearance rate before childbirth.

For infants: positive rates of HBV-DNA, HBsAg and HBeAg within 12 months postpartum.

### Data collection

1. Demographic information: age, gestational weeks, and initiation time of antiviral therapy of mothers; gestational age, body length, weight, head circumference, and Apgar score of infants.
2. Laboratory parameters: HBeAg, HBV-DNA before treatment, ALT, AST, serum creatinine, and estimated glomerular filtration rate (eGFR) at baseline and prior to delivery. Postpartum parameters include ALT and AST at 3 months and HBV-DNA at 6 months. HBV-DNA, HBsAg and HBeAg level of infants.
3. Pregnancy-related adverse events: preterm birth, dystocia, premature rupture of membranes, preeclampsia, postpartum hemorrhage, etc.
4. Adverse drug reactions: headache, muscle pain, nausea, fatigue, rash, etc.

### Statistical analysis

Statistical analysis was performed by Stata 17 software and graphs were plotted by GraphPad Prism 9.0. Normally distributed data were described as mean ± standard deviation (X ± SD) and analyzed using independent sample t-tests. Non-normally distributed data were expressed as the median (25th percentile, 75th percentile) and compared with the Wilcoxon rank-sum test. Qualitative data were presented as frequencies (n) or percentages (%) and analyzed using chi-squared tests or Fisher’s exact tests. *P* < 0.05 was considered statistically significant.

## Results

### Patients and general data

A total of 290 pregnant women meeting the criteria were included in the study, with 185 in the TDF group and 105 in the TAF group. Age, antiviral therapy initiation time, HBeAg positivity rate, and baseline levels of HBV-DNA, serum creatinine, and eGFR were not statistically different between the two groups (Table 1).

**Table 1:** Baseline data of patients in the two groups

Indicators	TDF group	TAF group	*P* value
	(*N* = 185)	(*N* = 105)	
Age (years), ®±S	30.10 ± 3.99	30.01 ± 3.78	0.952
Gestational time at delivery (days), M (Q1, Q3)	252(245,259)	245(245,252)	< 0.001
HBV DNA(Log10 IU/ml), M (Q1, Q3)	5.98(5.41,7.01)	5.72(5.48,7.34)	0.519
HBeAg positivity rate	87.50% (161)	88.57%(93)	0.788
Antiviral initiation time (weeks), M (Q1, Q3)	28.00(24.00,28.00)	28.00(24.00,28.00)	0.817
ALT (ratio to ULN), M (Q1, Q3)	0.80(0.58,1.18)	0.78(0.88,1.28)	0.015
AST (ratio to ULN), M (Q1, Q3)	0.81(0.63,1.18)	0.93(0.82,1.11)	0.004
Serum Creatinine (µmol/L), M (Q1, Q3)	57.00(50.10,62.50)	57.00(50.93, 62.93)	0.485
eGFR (ml/min), M (Q1, Q3)	130.04(116.56, 150.53)	130.46(115.91, 148.11)	0.627

($\bar{X}$ ±S)indicates Mean ± standard deviation M(Q1,Q3) indicates median(25th percentile, 75th percentile), ALT: Alanine transaminase, AST: Aspartate aminotransferase, ULN: Upper limit of normal, eGFR: Estimated glomerular filtration rate

### Treatment efficacy between the TDF group and TAF group

#### Effectiveness in mothers

**Fig. 1:**
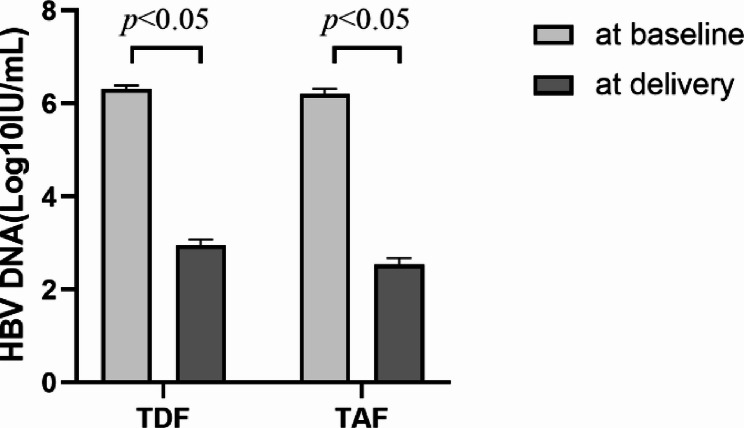
HBV DNA level at baseline and at delivery

HBV DNA levels before delivery in the TDF and TAF groups were 2.95 ± 1.80 Log_10_IU/ml and 2.53 ± 1.38 Log_10_IU/ml, respectively, and both showing significant decrease compared to baseline levels (*P* < 0.05) (Fig. 1). In addition, no significant differences were observed between the two groups in HBV DNA levels and seroclearance rates both at delivery and at 6 months postpartum. However, subgroup analysis revealed that at 6 months postpartum, those who continued treatment with TDF or TAF showed a significant decrease in HBV DNA levels compared to those who discontinued treatment at 1 month postpartum (all *P* < 0.001) (Table 2).

**Table 2:** Comparison of treatment efficacy of mothers

	TDF group				TAF group				*P*
	Total	Discontinuation (*n* = 130)	Continuation (*n* = 55)	P1	Total	Discontinuation (*n* = 78)	Continuation (*n* = 27)	P2	
HBV DNA (Log10 IU/ml) at delivery	2.95 ± 1.80	2.54 ± 1.66	3.91 ± 1.76	< 0.001	2.54 ± 1.38	2.59 ± 1.38	2.41 ± 1.39	0.562	0.132
HBV DNA clearance at delivery	29.19%				26.92%				
HBV DNA (Log10 IU/ml) at PPM6	3.56 ± 2.06	4.23 ± 1.93	1.98 ± 1.41	< 0.001	3.40 ± 1.67	3.89 ± 1.40	2.01 ± 1.60	< 0.001	0.998
ALT (ratio to ULN) at delivery	0.80 ± 0.40	0.83 ± 0.47	0.75 ± 0.26	0.783	1.03 ± 0.40	1.04 ± 0.44	1.02 ± 0.31	0.580	0.052
AST (ratio to ULN) at delivery	0.76 ± 0.29	0.77 ± 0.32	0.74 ± 0.22	0.272	1.16 ± 0.49	1.17 ± 0.51	1.15 ± 0.44	0.637	0.105

PPM: Postpartum months; ALT: Alanine transaminase; AST: Aspartate aminotransferase; *P*1: Discontinuation (treatment less than 1 month postpartum) vs. Continuation (treatment over 1 month postpartum) of TDF treatment; *P*2: Discontinuation (treatment less than 1 month postpartum) vs. Continuation (treatment over 1 month postpartum) of TAF treatment; *P*: TDF group VS TAF group

#### MTCT of HBV

In the TDF group, 185 pregnant women delivered 190 babies, including five sets of twins, while in the TAF group, 105 pregnant women delivered 106 babies, including one set of twins. Failure to prevent MTCT of HBV was defined as newborns testing positive for HBsAg, HBeAg, or HBV DNA > 10 IU/mL within 7–12 months after birth. In the TDF group, two newborns tested positive for HBsAg, one at 7 months and another at 12 months postpartum. In the TAF group, one newborn tested positive for HBsAg at 7 months postpartum. The comparison of HBV transmission rates showed no significant difference between the two groups (*P* = 1.000) (Table 3).

**Table 3:** Comparison of MTCT of HBV between two groups

	Seven months		Twelve months	
	TDF	TAF	TDF	TAF
HBsAg positive	1	1	1	0
HBeAg positive	0	0	0	0
HBV DNA positive	0	0	0	0
MTCT rate	TDF VS TAF: 1.08% vs. 0.95%, *P* = 1.000			

MTCT: Mother-to-child transmission

### Safety evaluation

#### Side effects of drugs

**Fig. 2:**
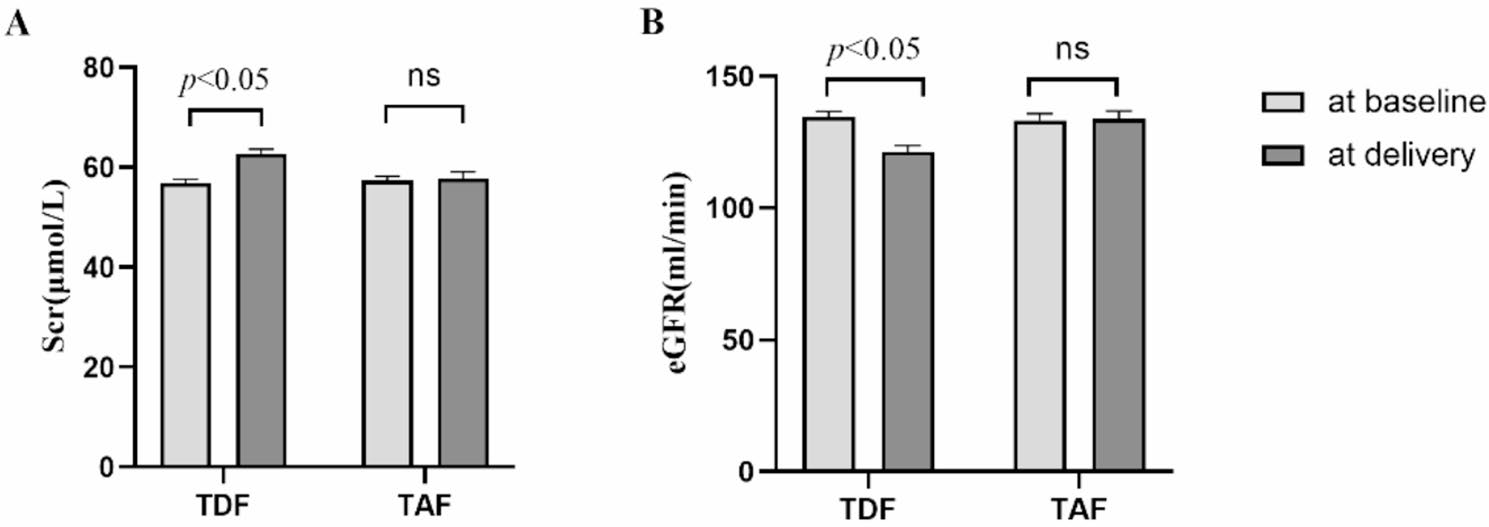
Comparison of renal function

During pregnancy, 3 patients in the TAF group and 7 patients in the TDF group experienced adverse reactions. All adverse reactions were manageable and didn’t require treatment interruption. The overall adverse reaction rates were no significantly different between the two groups (2.86% vs. 3.78%, *P* = 0.936) (Table 4). However, significant changes in serum creatinine and eGFR were observed after TDF therapy (*P* < 0.05), whereas no significant changes were detected in the TAF group (Fig. 2). This findings suggest a potential risk of kidney damage associated with TDF treatment.

**Table 4:** Comparison of adverse drug reactions between the two groups

	TDF group(*N* = 185)	TAF group(*N* = 105)	*P* value
Headache	1	1	1.000
Muscle pain	1	1	1.000
Rash	1	0	1.000
Nausea	3	0	0.556
Fatigue	0	1	0.362
Nasal hematoma	1	0	1.000
Rate of adverse drug reactions	3.78% (7/185)	2.86% (3/105)	0.936

#### Adverse pregnancy outcomes

15 adverse pregnancy-related events occurred in the TAF group, including 5 preterm births,1 postpartum hemorrhage (PPH), 2 placenta accreta and 7 premature rupture of membranes (PROM). In comparison, 22 adverse pregnancy events occurred in TDF group, including 8 PROM, 8 preterm births, 1 dystocia, 3 placenta accreta, 1 PPH, and 1 eclampsia. The overall incidence of adverse pregnancy-related events was not significantly different between the two groups (*P* = 0.557) (Table 5).

**Table 5:** Comparison of adverse pregnancy outcomes between the two groups

	TDF group(*N* = 185)	TAF group(*N* = 105)	*P* value
Eclampsia	1 (0.54%)	0	1.000
PROM	8 (4.32%)	7(6.67%)	0.387
Preterm birth	8 (4.32%)	5 (4.76%)	0.863
Dystocia	1 (0.54%)	0	1.000
Postpartum hemorrhage	1 (0.54%)	1 (0.95%)	1.000
Placenta accreta	3(1.62%)	2(1.90%)	1.000
Overall incidence	22(11.89%)	15(14.29%)	0.557

PROM: premature rupture of membranes

#### Congenital abnormalities and neonatal growth percentiles

All newborns (190 in the TDF group and 106 in the TAF group) were healthy with no birth defects. There were no significant differences in body length, head circumference, birth weight, gestational age and Apgar scores at birth between the two groups (*P* > 0.05) (Table 6). Additionally, no growth or developmental abnormalities were observed in either group.

**Table 6:** Comparison of growth and developmental abnormalities in newborns

	Newborns in TDF group (*N* = 190)	Newborns in TAF group (*N* = 106)	*P* value
Birth weight (n, %)			0.696
2500 g ≤ and ≤ 4000 g	11(5.79%)	5(4.72%)	
< 2500 g or > 4000 g	179(94.21%)	101(95.28%)	
Body length (cm)	50.07 ± 0.65	49.95 ± 1.08	0.330
Head circumference (cm)	34.02 ± 0.18	33.99 ± 0.24	0.809
Gestational age (n, %)			0.955
< 37w	14 (7.37%)	8 (7.55%)	
≥ 37w	176(92.63%)	98(92.45)	
Apgar score	9.48 ± 0.83	9.48 ± 0.933	0.711

## Discussion

CHB, caused by HBV, is a common infectious disease, with MTCT being a primary route of spread. Both TAF and TDF, prodrugs of tenofovir, are first-line antiviral drugs for hepatitis B [15]. TDF is recommended as the first choice for pregnant women in guidelines. TAF, as a next-generation prodrug, offers similar antiviral efficacy to TDF with improved renal and bone safety [16]. However, previous studies have not specified whether mothers had prior antiviral drugs, whereas this study focuses on treatment-naïve mothers to evaluate the efficacy and safety in MTCT.

According to the Guidelines for the Prevention and Treatment of Chronic Hepatitis B (2022 version), the immune-tolerant and immune-clearance phases are defined. It is important to note that while most patients in these phases are HBeAg-positive, this serostatus does not universally indicate the immune-tolerant phase. Although the majority of the patients included in the study were HBeAg-positive, this doesn’t mean that they were all in the immune tolerance phase. Some patients may be in the immune clearance phase, so the baseline HBV DNA level is relatively low.

High HBV DNA level is closely related to failure in blocking MTCT and immune prophylaxis [4, 17]. In our study, the baseline HBV DNA levels (approximately 6 log₁₀ IU/mL) were lower than expected [18]. This may be attributed to the heterogeneity in the immune phases of the cohort, as many participants were not in the immune-tolerant, HBeAg-positive phase. Physiological changes during pregnancy may also influence HBV DNA levels, resulting in lower viral loads than those seen in the immune-tolerant phase. Antiviral treatment during pregnancy reduces HBV loading, lowering the risk of MTCT [19]. Previous studies showed that TAF and TDF were highly effective in MTCT prevention [20, 21]. In this study, we showed that TAF is as effective as TDF in reducing viral load in treatment-naïve mothers with high viremia, which was consistent with previous studies. Additionally, Wen et al. reported no HBsAg-positive infants within one year after birth when mothers with high viral loads received TAF treatment in the late stages of pregnancy [22]. Similarly, the low MTCT rate was observed after TDF and TAF treatment in our study. These results suggested that TAF and TDF are effective in preventing HBV MTCT in treatment-naïve mothers.

Interestingly, we observed that HBV DNA levels did not return to baseline at PPM6 in both TDF and TAF group. This may be due to enhanced immune control following postpartum immune reconstitution, which can suppress HBV replication [23]. Additionally, the sustained suppressive effect of antiviral treatment on cccDNA transcription could delay viral rebound [24]. It is also possible that immune-mediated viral suppression occurred without overt biochemical flare. Given that viral rebound may take longer than 6 months, extended follow-up is necessary to fully understand the long-term dynamics of HBV post-treatment cessation.

TAF and TDF are generally well-tolerated, with mild side effects such as headache, nausea, rash, fatigue, and muscle pain. In our study, side effect rates in the TAF and TDF groups were 2.86% and 3.78%, respectively, and all the effects were managed without discontinuing treatment. Additionally, adverse pregnant events were also low with 15.24% in the TAF group and 12.43% in the TDF group, consistent with previous studies showing no increased pregnancy complications [12, 22–26]. However, TDF carries a potential risk to the kidney damage [27–29], and our results also found increased serum creatinine and decreased eGFR after TDF treatment. TAF, by contrast, demonstrated a significantly lower risk of kidney damage.

We propose that the following factors may explain why HBV DNA levels did not return to baseline six months after delivery. First, owing to the inherent limitations of this single-center retrospective study, including its limited sample size, the 6-month follow-up period may have been insufficient to observe complete viral rebound after treatment cessation, and selection bias cannot be excluded. Second, we think short-term antiviral therapy could contribute to sustained viral suppression after cessation through the mechanism of partially restored cell function, thereby enhancing immune control. This thought is consistent with the existing literature. Studies have shown that discontinuing tenofovir during childbirth does not increase the risk of virological recurrence, clinical recurrence or retreatment, highlighting the sustained inhibitory effect of antiviral drugs.

Newborns, with their developing immune systems, are particularly vulnerable, making the potential effects of medications on their growth, development, and risk of birth defects a major concern. Toxicology studies have shown that TDF and TAF do not interfere with DNA synthesis and are not associated with birth defects or mutations. Clinical studies have also confirmed the safety of TDF and TAF during pregnancy, with no reports of birth defects and growth or developmental issues in infants [30, 31]. In our study, no congenital defects were observed in either group, and infants in the TAF group showed no significant differences in growth and development compared to the TDF group. The birth weight and gestational age of the newborn were basically in the normal ranges. This is consistent with previous research [32]. These findings suggest that TAF and TDF are safe for newborns, though long-term observations are recommended to further confirm the results.

In summary, this study highlights that both TAF and TDF are effective in preventing MTCT of HBV during pregnancy in treatment-naïve mothers. However, TAF demonstrated superior renal safety compared to TDF. These findings provide additional evidence for the use of TAF in preventing perinatal HBV transmission in treatment-naïve mothers.

This study has certain limitations. Firstly, it is a single-center retrospective study with a relatively small sample size and a limited follow-up period. Secondly, a more comprehensive comparison of the safety between TAF and TDF could benefit from additional parameters, such as bone density, serum calcium and phosphate, lipid profiles, and renal tubular function. To further validate the effectiveness and safety of TAF in preventing MTCT of HBV, multi-center studies with lager sample sizes, longer follow-up periods, and more extensive evaluation criteria are needed.

## Data Availability

The data is from the outpatient system of Xiangya Hospital. For ethical, privacy or security reasons, the patient’s name and outpatient number cannot be shared. The original datasets presented in the study are available from the corresponding author on reasonable request.
